# Towards remote assessment and screening of acute abdominal pain using only a smartphone with native accelerometers

**DOI:** 10.1038/s41598-017-13076-x

**Published:** 2017-10-06

**Authors:** David R. Myers, Alexander Weiss, Margo R. Rollins, Wilbur A. Lam

**Affiliations:** 1 The Wallace H. Coulter Department of Biomedical Engineering, Georgia Institute of Technology & Emory University, Atlanta, GA 30332 USA; 2 Department of Pediatrics, Division of Pediatric Hematology/Oncology, Aflac Cancer Center and Blood Disorders Service of Children’s Healthcare of Atlanta, Emory University School of Medicine, Atlanta, GA 30423 USA; 3 Winship Cancer Institute of Emory University, Atlanta, GA 30423 USA; 4 Parker H. Petit Institute of Bioengineering and Bioscience, Georgia Institute of Technology, Atlanta, GA 30332 USA

## Abstract

Smartphone-based telehealth holds the promise of shifting healthcare from the clinic to the home, but the inability for clinicians to conduct remote palpation, or touching, a key component of the physical exam, remains a major limitation. This is exemplified in the assessment of acute abdominal pain, in which a physician’s palpation determines if a patient’s pain is life-threatening requiring emergency intervention/surgery or due to some less-urgent cause. In a step towards virtual physical examinations, we developed and report for the first time a “touch-capable” mHealth technology that enables a patient’s own hands to serve as remote surrogates for the physician’s in the screening of acute abdominal pain. Leveraging only a smartphone with its native accelerometers, our system guides a patient through an exact probing motion that precisely matches the palpation motion set by the physician. An integrated feedback algorithm, with 95% sensitivity and specificity, enabled 81% of tested patients to match a physician abdominal palpation curve with <20% error after 6 attempts. Overall, this work addresses a key issue in telehealth that will vastly improve its capabilities and adoption worldwide.

## Introduction

Enabling patients to receive a medical diagnosis and treatment when geographically separated from a physician has been a long-standing goal of healthcare, as exemplified by telemedicine and the direct-to-consumer telemedicine industry. Preliminary^1^, evidence indicates that telemedicine approaches reduce physician time spent on consults^2^ and curb demand on facilities^3^, using stretched resources more efficiently. Furthermore, telemedicine enables patients to receive medical advice from the comfort of their home, which is especially helpful those living in a medically underserved community. Such communities are common in both developed and resource poor settings, for example 20% of Americans^4^ live in a medically underserved area as defined by too few and/or geographically distant clinics. However, telemedicine-based approaches suffer from the inability of physicians to gather the same information available in clinical settings. For example, evaluations of telemedicine services found less diagnostic testing and subsequent poorer performance in correctly assessing and prescribing or avoiding antibiotics for conditions such as pharyngitis (sore throat) or bronchitis, conditions that benefit from diagnostic testing^5^.

Smartphone-based mobile health (mHealth) technologies have enormous potential in addressing deficiencies with current telemedicine approaches by analyzing, processing, and communicating data from a suite of sensors able to assess health^6–8^. Medical grade imaging and new point-of-care diagnostics capably replicate some tests performed in clinics^9–13^. Moreover, smartphones are widely available throughout the world and used by 69% of individuals in developed countries as well as 46% of populations in developing countries^14^. Noting the highly developed cellular networks in developing countries, mHealth technologies may help leapfrog over traditional barriers to accessible medical care. However, while current mHealth technologies do provide additional point of care capabilities, they are still unable to substitute for the physical examination, which is the most common type of patient and physician interaction.

Modern medicine relies on medical encounters that primarily consists of two components: a patient history and physical examination^15^. During such an exam, a physician: inspects, feels (palpates), taps (percussion), and listens (auscultation) to the patient. Advances in mHealth technologies have enables some of these actions to be done from afar, for example visualizing^16,17^, or listening to the patient^18^. However, approaches enabling physicians to remotely palpate on patients are conspicuously absent, yet needed to fully enable remote physical exams. Here, our initial focus lies in creating a tool useful for triage, which is different from diagnosis as it has a different end goal. The medical triage process involves the sorting of patients (as in an emergency room) according to the urgency of their need for care, whereas diagnosis seeks to understand the root cause of a patient’s ailment. In triage, a clinician first assumes that the patient has a life threatening condition and will subsequently seek evidence to the contrary. For patients complaining of acute abdominal pain, a physician will first suspect that a patient has appendicitis and will then seek evidence indicating otherwise, specifically by using a physical examination and palpations. Severe tenderness with palpations strongly indicates that the patient should seek immediate treatment.

Enabling remote palpations for triage could dramatically reduce demand on emergency departments. Approximately 10% of all emergency department visits relate to abdominal pain^19^, but of all these visits, only 1 in 10 required urgent attention^20^. Therefore, 9 of 10 cases related to abdominal pain or 9% of all emergency department visits, did not need urgent attention and might have been treated in less expensive venues. The challenge has been creating a technique that enables physicians to perform triage for abdominal pain from a geographically separated location. To that end, we enable remote interactions with a new technique that trains a patient’s own hands to palpate and act as a surrogate for a physician. Since this technique leverages the innate ability of modern smartphones to track movement, no additional hardware or devices other than a smartphone are needed.

## Results

A variety of sensory inputs such as heat, touch, pressure, textual information, and motion guide a physician performing palpations on a patient. This research seeks to address whether motion information gathered from a single axis accelerometer is sufficient to provide useful information that enables physicians to screen and triage abdominal pain, that is, examine and assess the urgency of patient need. As the first study to report the use of the motion tracking capabilities of a cellular phone towards assessing abdominal pain, this work also seeks to identify standards for proper palpation using a phone. Results from this approach would be broadly applicable as nearly all current smartphones have at least one accelerometer.

**Figure 1:**
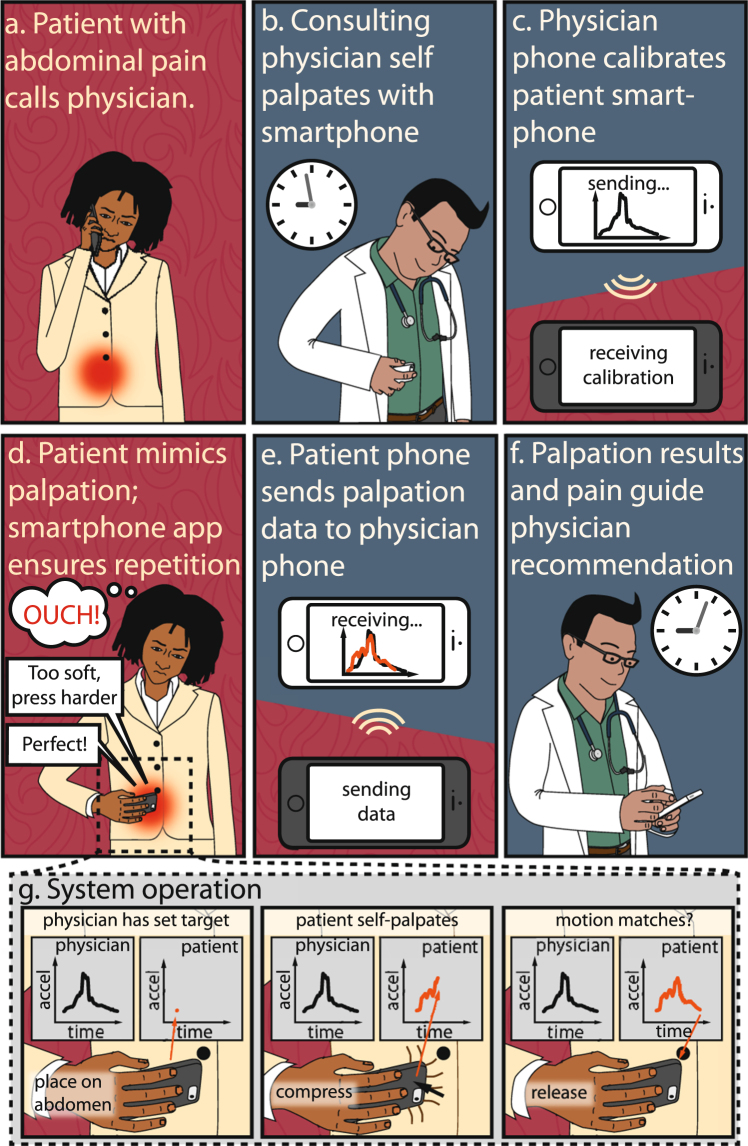
Smartphones with accelerometers enable physicians to remotely screen patients with abdominal pain and refer them to the appropriate medical facilities. (**a**) An individual calls a physician complaining of abdominal tenderness and wants to know if they should seek further medical evaluation. (**b**) After taking a medical history, the on call consulting physician self-palpates and the smartphone accelerometer records the movement. (**c**) The physician then uses this movement to calibrate an application on the patient’s smartphone and begins a video based meeting with the patient. (**d**) The patient launches the app and self-palpates using the integrated instructions and real-time feedback to ensure the palpations match physician calibrated numbers. While the patient performs self-palpations, the physician watches the patient to monitor both technique and pain levels. (**e**) The app will sends the patient palpation data to the physician to ensure that the palpation matches the physicians calibration (**f**) Based on the medical history and physical examination, the physician is able to refer the patient to the best care. (**g**) By using integrated accelerometers to quantitatively measure the patient’s palpation motion, it is possible to compare the patient and physician’s motion as well as provide feedback to the patient on how to better match the physician.

In our proposed implementation, a patient with abdominal pain contacts a physician (Fig. 1a) to determine if they should seek further medical evaluation. A physician begins the remote medical exam by evaluating the patient’s symptoms and medical history. If remote palpations are needed, the physician calibrates the system by performing a self-palpation using their own smartphone (Fig. 1b). Integrated micro-electro-mechanical accelerometers measure the motion of the physician palpation, and the resulting data is transmitted over the cellular network to calibrate the patient’s smartphone (Fig. 1c). The physician then initiates a video call with the patient to monitor their palpation technique and to visually assess pain levels. After starting an application on their smartphone, the patient receives instruction on palpation technique and performs a self-palpation using the smartphone. The smartphone application measures the patient self-palpation, compares it to the physician palpation, and provides feedback as to how to match the physician palpation: more or less depth, and shorter or longer compression (Fig. 1d). Once a patient matches the physician palpation, the physician receives the patient data (Fig. 1e), enabling the physician to quantitatively verify that the patient matched their own palpation (Fig. 1f). Using both information on the palpation and on the patient pain levels, the physician determines how urgently care is needed and recommends an appropriate venue: emergency care, primary care, or home based health remedies with a follow-up. Our results focus on showing the feasibility of this system by demonstrating: that smartphone motion measurements are precise and accurate; that physicians and patients may self-palpate with a smartphone; and that our feedback application enables physician palpations to be repeated by patients.

**Figure 2:**
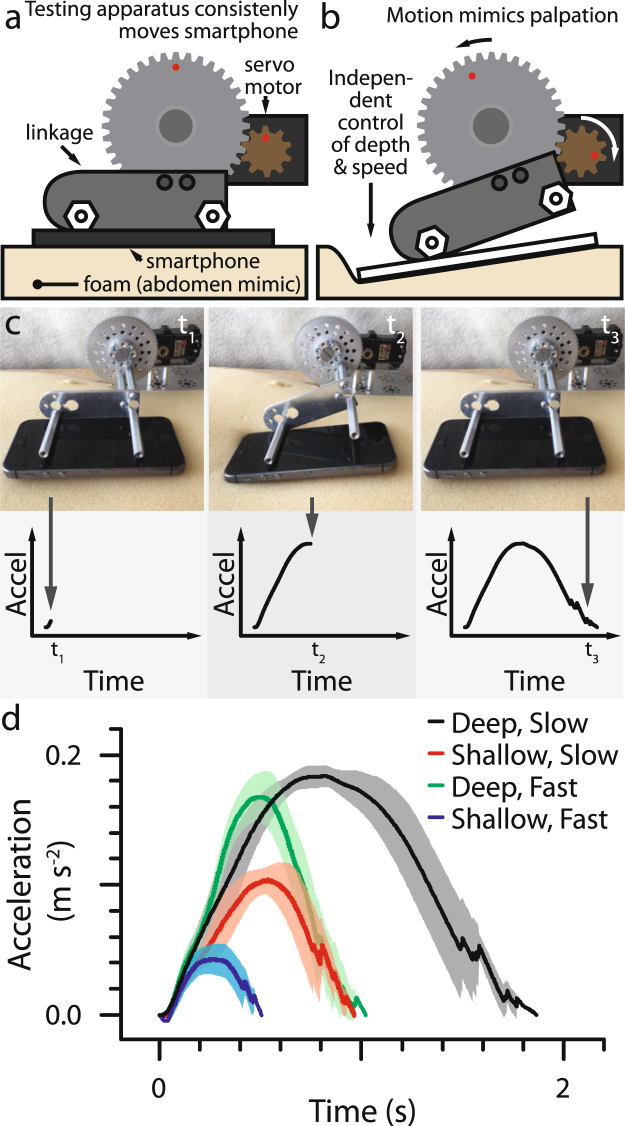
Phone accelerometer resolves differences in palpation motion. (**a**,**b**) To test the repeatability of the smartphone, the acceleration was recorded while repetitively moving the smartphone into an abdomen like structure (foam) using a precise servo motor (**c**). At rest, the acceleration is zero and steadily increases while the phone is pushed into the abdomen until reaching the maximum depth. As the phone returns to the initial position, the acceleration decreases and returns to zero. (**d**) The single-axis accelerometer correlates with motion and shows that a variety of palpation speeds and depths are distinguishable. Each condition was repeated eight times.

Our data indicates that a smartphone with a single axis accelerometer has sufficient accuracy and precision to measure the motion associated with a self-palpation. After examining a board-certified physician apply self-palpations, we constructed an apparatus to apply similar motions to a model of the abdomen in a controlled and reproducible manner. A servo motor powered gearbox (Fig. 2a–d) rotates a linkage that applies palpations with a smartphone to a piece of upholstery foam (Fig. 2a–d). The palpation depth and speed are controlled by setting the angle of rotation and velocity, respectively, on the servo motor. Since this device will be used by the medically untrained public, who are expected to have more varied self-palpations, a wide range of depths and speeds were tested. Each of the tested compressions are clearly distinguishable from each other and repeatable as indicated by the small standard deviation and lack of hysteresis (Fig. 2e). In the phone-abdomen system, the position, velocity, and acceleration are inter-related and measuring or describing any one of these quantities will be sufficient to define the motion of the object. While this report presents all findings in terms of acceleration, descriptions of position and velocity are included when appropriate, as they are more intuitively accessible (See Methods). Overall, our result confirms that the smartphone precisely resolves different compression depths and speeds associated with an abdominal palpation motion.

Upon translating this method to use with patients and physicians, we determined that an appropriate use protocol minimized the collection of spurious or incorrect data. A number of factors influence the acceleration data for each palpation including: the palpation movement, gravity, small rotational changes, and high-frequency noise. In addition, the acceleration data may include measurements of movement that are not associated with the actual palpation, such as acceleration data recorded while bringing the phone to the correct anatomical location or other non-desired movements. Recognizing and removing such spurious movement data with an algorithm is difficult and computationally intensive with information from only a single axis accelerometer. Instead, we found that asking the user to hold the smartphone still before and after remote abdominal palpations had the effect of eliminating the collection of unwanted data associated with non-desired phone movement. In this approach, a user rests the smartphone on their abdomen in the same physician specified position both before and after a palpation. This step inserts a period of zero acceleration between the relevant palpation curves that was identified and removed by the data refinement algorithm. This protocol removes nearly all sources of erroneous acceleration data with the exception of inadvertent rotation, which may reduce the signal amplitude. To minimize rotation errors that reduce signal amplitude, the protocol aligns the accelerometer measurement axis with the desired measurement direction. Based on an analytical model, moderate rotations (19 degrees) introduce a relatively small error (5%) (Supplemental Fig. 1). Therefore, small rotations will have a minimal effect on the data, while large rotations will be visible to the observing physician, who may offer advice to improve the patient’s technique.

In combination with the use protocol, the refinement algorithm transforms the raw data into a form that facilitates comparisons of the acceleration magnitude and duration. The algorithm uses a series of sequential operations and begins by removing constant offsets, which may be attributed to a constant gravitational force (Supplemental Fig. 2b). Next, zero values that are associated with no phone movement are removed (Supplemental Fig. 2c). Finally, a moving average filter (See Methods) is applied (Supplemental Fig. 2d), and the curve is transformed to end on zero (Supplemental Fig. 2e). The final transformation accounts for situations where the smartphone ends the palpation in a slightly different orientation. In this case, data is assumed to be a superposition of the desired palpation curve and an error associated with the rotation of the phone in gravity. It is further assumed that the error associated with the rotation of the phone in gravity occurred steadily and linearly over the duration of the palpation. Hence, the user did not rotate the phone suddenly at the end of the palpation, but gradually and steadily applied the undesired rotation when palpating. In this case, the rotational error is approximated as a linearly increasing offset and subtracted from the data. Taken together, the instructions to the user and data refinement algorithm produce data that is easy to compare and contrast.

**Figure 3:**
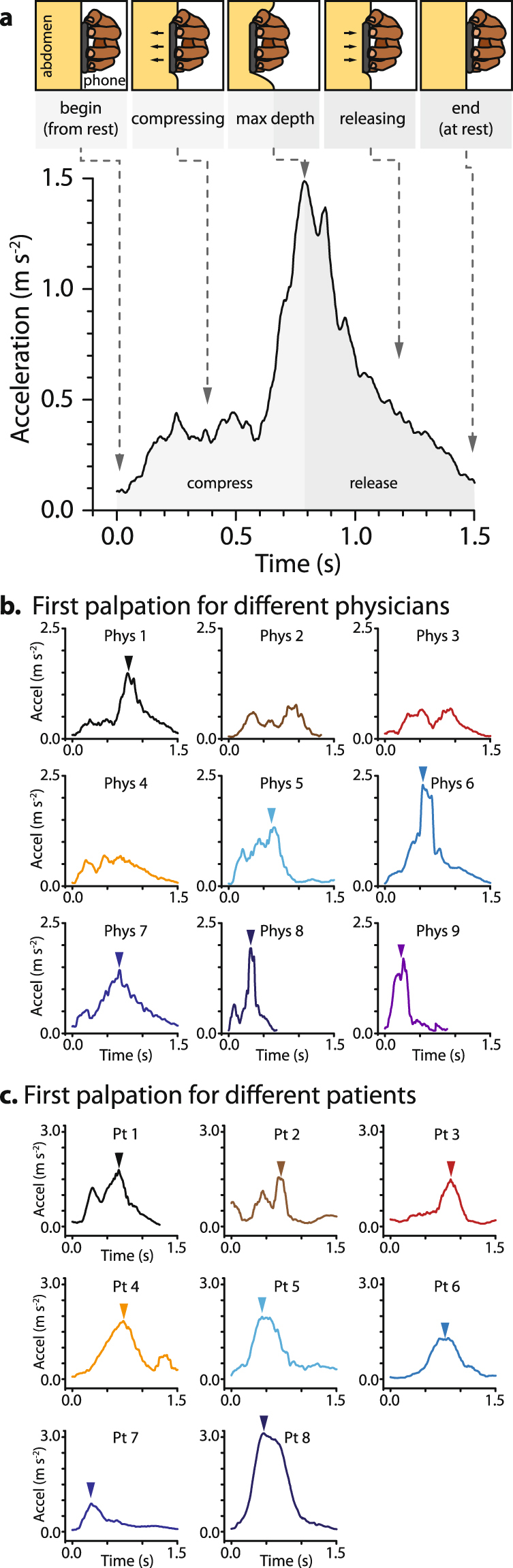
Smartphone measured self-palpations are broadly similar among physicians and patients. (**a**) The individual places the phone on their abdomen and palpates using a motion similar to that used with hands alone. Although the acceleration curve seen with the servo-motor also contained a single peak, the rate of acceleration increases faster when performed with a hand on an actual abdomen. (**b**) Physicians exhibit broadly similar curve shapes, indicating that each physician uses similar motions with the new smartphone system. (**c**) The first recorded patient self-palpation curves tend to have a single peak in the same timeframe with a slightly higher range of magnitudes than physicians. This indicates that the self-palpation motion is intuitive and accessible to non-medically trained individuals. In (**b** and **c**), the triangle indicates the location of the peak. In cases where multiple peaks are present, the individual would be asked to use a single peak palpation style.

Both physicians and patients successfully use the smartphone based accelerometry system to measure abdominal self-palpations. Patient and physician self-palpation curves contain a clear increase, peak, and subsequent decrease (Fig. 3a), as would be expected from a compression and subsequent release on the abdomen. Beginning at time 0 with the hand and phone at rest, the acceleration increases until reaching a maximum that coincides with the maximum depth. Upon release, the acceleration falls and returns to zero. While, the compression and release shape match that of the testing apparatus (Fig. 2c), the acceleration is less smooth and typically increases and decreases more rapidly around the peak. Both the use of an actual abdomen and performing hand guided palpations likely contribute to the observed difference from the testing apparatus.

Physicians seek to perform consistent palpations to improve abdominal pain assessment. In a small survey of four physicians, all strongly agreed with the statement: “Abdominal pain assessment is better when a provider uses consistent palpations from patient to patient as opposed to when a provider uses inconsistent palpations from patient to patient”. To test physician consistency, one physician was asked to perform repeated self-palpations using the smartphone (Supplemental Fig. 3). Without feedback, the physician tended to switch between two palpation styles, one defined by a single peak and one defined by multiple peaks, with both occurring in a similar timeframe. Although the physician switched between styles, key features of each palpation style were consistent. In the single peak style (palpations 2,4, and 6) the maximum magnitude and standard deviation were 1.55 m/s^2^ and 0.071 m/s^2^, while the average and standard deviation of the time from rest to peak acceleration were 0.30 s and 0.051 s. In the multi-peak style, while the time was not as consistent, the maximum magnitude was with an average and standard deviation of 0.989 m/s^2^ and 0.079 m/s^2^. Hence, this data supports the idea that a physician may switch between palpation styles, but remain consistent when using a specific style.

However, while physicians seek to perform consistent palpations, they have small and unique stylistic differences from one another. Here, 9 physicians were given the smartphone, and when ready, their first palpation was recorded. The majority of palpation curves exhibited a single clearly defined peak with a rapidly increasing compression (Fig. 3b), although approximately 33% of the physicians peaks with a low magnitude (Physicians 2–4). Each physician has their own distinct style of palpation as measured by variations in magnitude, duration, and rate of increase around the peak. For example, physicians 8 and 9 use fast deep palpations, and physicians 1, 6, and 7 prefer more shallow and slow palpations. Each of these physicians uses their own unique signature palpation style to assess a patient’s tenderness and pain. As such, a key need to facilitating a long distance interaction between a patient and a physician is exactly recapitulating the physician’s own palpation curve remotely.

Our data on patient self-palpations (Fig. 3c), supports the idea that non-medically trained personnel with minimal guidance are able to move in a motion similar to a physician. Here, 4 male and 4 female patients between the ages of 20–35 were given the smartphone with minimal instruction (See Methods: Volunteer Testing), and when ready, the first palpation was recorded. Like the majority of physicians, all patient self-palpation curves contain a single acceleration peak. In addition, as the patients exhibited a range of peak accelerations and timeframes that span the range covered by the physicians, this suggests that patients have the necessary manual dexterity to repeat the physician’s motion.

To quantitatively compare the patient and physician palpation curves, the impulse of the cell phone was calculated using the acceleration data and mass of the cellphone (145 g). While the qualitative assessment examined the shape of the curves (single vs multi-peak), this quantitative assessment helped identify the energy placed into the palpation movement regardless of specific style. Our goal in using the broad average was to illustrate that the specialty training received by a physician does not significantly increase the palpation energy and therefore suggest an increase in strength or dexterity when compared to an untrained individual. Since normality cannot be rejected by the Shapiro-Wilk test, an unpaired student t-test was used to compare the physician and patient impulse. The average physician impulse and standard deviation of 95.4 mN·s and 31.96 mN·s (n = 9) is not significantly different from the patient impulse of 145.86 mN·s +/−62.7 mN·s (n = 8). However, as the p-value is 0.0504, further testing may conclude that patients have higher impulses than physicians. Regardless of whether the patients have statistically equal or higher impulse values, this data supports the idea that patients possess sufficient dexterity and strength to repeat the self-palpation motion of a physician. This implies that the primary challenge in enabling patients to match the physician palpation lies in providing guidance and feedback to the patients as to how their own palpation curves differ from physicians and not that the patients are physically incapable of achieving the same palpation curves as physicians.

We created a smartphone application (app) to provide feedback that enables an individual to match a pre-specified palpation. Providing a mechanism for feedback is key to enabling an individual to match a target palpation quickly. This is especially true when the target palpation is stylistically different in timing or magnitude from the individual’s typical palpation. To use the application, a physician sets a target ideal palpation curve. As the majority of physicians and patients exhibited a palpation style defined by an increase in acceleration to a single peak, then a subsequent decrease to zero, this work focuses on matching these palpation curves between individuals. In the current iteration of our smartphone app, a physician who used a palpation style that does not contain a single peak is asked to adjust his or her palpation to the single peak style. Our examination of a single physician performing repeated self-palpations revealed that a physician switched between two distinct styles in which a single peak and multi-peak palpation style is used (Supplemental Fig. 3) which therefore suggests that physicians have some flexibility in the palpation style used. In addition, as our survey indicates that physicians strive to use consistent palpations during their abdominal exams, future versions of the application will ask the physician to perform multiple palpations and review these for consistency before setting the ideal.

**Figure 4:**
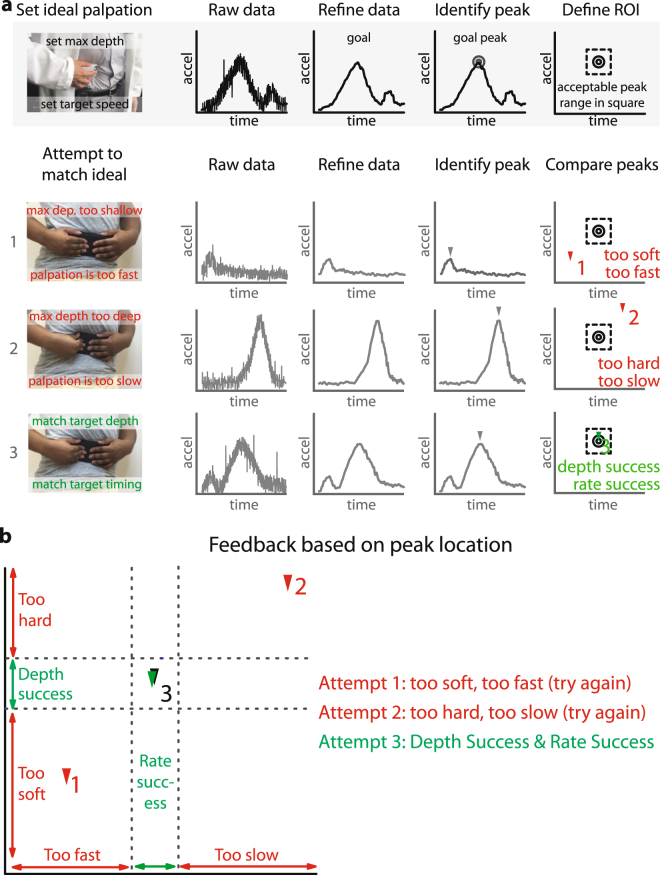
Application compares curves to ideal palpation and provides feedback after each matching attempt. (**a**) To use the feedback application, a physician sets an ideal curve or goal based on their own preferred palpation style. The raw data is filtered by the refinement algorithm and the application identifies the peak acceleration magnitude and timing. If a matching peak occurs within a set window (here 20%) of the ideal peak, then the palpation matches. In the illustration above, the individual’s first attempt is too soft and too fast, with the peak occurring to the left and below the target peak. The second attempt is too hard and too slow, with the peak occurring above and to the right of the target peak. The third match is successful in matching both the time and magnitude of the initial peak. (**b**) Feedback is provided to the individual based on the location of the attempted peak relative to the ideal peak.

After self-palpating, the data refinement algorithm then transforms the raw data into a form that facilitates comparisons (Fig. 4a). This feedback application identifies the peak and a region of interest or acceptance window, which here is defined as a percentage of the peak magnitude and time. Hence, when a physician performs a self-palpation, the application identifies the duration and magnitude of the compression portion of the palpation for subsequent comparison. More specifically, the feedback application will compare the physician’s time from rest to peak acceleration as well as the magnitude of the peak acceleration. Since this is the first reported study to use smartphone-based palpations, it is currently unclear what acceptance window is sufficient for screening of acute abdominal pain. However, before performing clinical studies to determine an optimal acceptance window, we sought to establish whether the feedback application enabled patients to match physician palpations. Noting that the standard deviation of the peak magnitude of one physician’s repeated single peak palpations was 4.6% and that the standard deviation of the time was 17%, we hypothesized that the medically untrained public could achieve a motion within 20% of the magnitude and time of the physician. This acceptance window was chosen based on several factors, including considerations of how well physicians may repeat motions, and providing a balance to the patient between ease of use and fidelity to the physician specified motion.

After the ideal palpation is set, a patient performs matching attempts by following the on-screen instructions. The data refinement algorithm transforms the raw data from the attempt and the feedback application identifies the associated peak magnitude and timing. If the attempted palpation peak is within the acceptance window of the ideal peak, then the attempt successfully matched the ideal palpation. In any other case, the feedback application determines the relative difference between peaks and provides suggestions on how to better match the ideal (Fig. 4b). Initial tests show that non-medically trained individuals were able to repeat their own target abdominal palpation curve using the feedback application (Supplemental Fig. 4a). By choosing a simple metric for feedback, such as the peak timing and position, plain and intuitive instructions are relayed to the individual on how to better match the target palpation. However, this simplicity allows for minor changes in the curve shapes to be present (Supplemental Fig. 4b). This highlights the need for physician judgement on whether the patient’s self-palpation was sufficient or should be repeated. For example, some patients exhibited a single peak palpation motion that has a different peak width than that of the physician. This variation in matching curves occurs because the algorithm measures the peak magnitude and the time from rest to peak, but not the rate at which the peak magnitude was reached. As such, the physician would need to review the motion data of the patient to decide whether the matching peak indicated by the app met his or her own criteria for a match.

**Figure 5:**
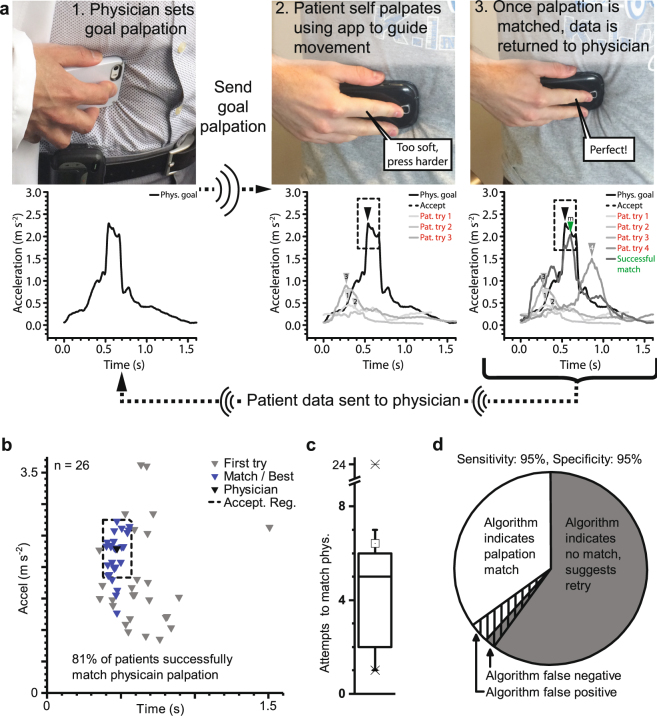
Patients match physician specified palpations from a remote location using the feedback algorithm. (**a**) The physician uses their smartphone to palpate using their preferred depth and duration. This calibration curve is sent to the patient’s smartphone which then provides feedback after each patient self-palpation to enable the patient to match the physician specified curve. In the first three attempts, the patient palpation is too short and not of an adequate depth. Once the patient palpation matches the physician specified palpation, the data is returned to the physician for review. Here the matching patient compression is not as fluid and smooth as the physician curve, but the depth, duration, and release are similar. (**b**) The algorithm effectively provided feedback that enabled patients to alter their native palpation style to match that of the physician in 85% of cases, while all 100% of cases seeing some improvement in style (n = 26). (**c**) A median number of 5 attempts were needed by the patients that successfully matched the physician palpation curve. (**d**) The high sensitivity and specificity (n = 57) of the application ensures that patient receives effective feedback instructions. Since false positive and negative curves may typically be recognized as highly unorthodox shapes, the supervising physician may focus on the patient and not on scrutinizing each peak.

With the completed use protocol, data refinement algorithm, and integrated smartphone application, we demonstrated that a physician may set a palpation that a patient repeats in a geographically different location. Here, a physician set the target abdominal palpation (Fig. 5a) and then transmitted the information to an individual for remote testing. For the patient shown in Fig. 5a, the first two individual palpations were too fast and shallow. As the application provided feedback, the palpations become deeper, but the individual slightly overcompensated on the timing before completing a matching palpation (Fig. 5c). The physician then received the patient data to ensure that the patient palpation indeed matched the target palpation. In our small pilot study of 26 healthy patients, we determined that the native smartphone hardware and feedback algorithm enabled the vast majority of patients to match a physician specified palpation. Here, 12 males and 14 females between the ages of 20–35 were recruited for the study. Patients using the smartphone and feedback algorithm were given up to four sets of nine attempts to self-palpate palpate to within 20% of a physician specified curve. Noting that feedback cannot be given until after the first palpation, the first palpation serves as a good indicator of the patient’s preferred palpation depth and time using the smartphone. Of the study participants, only 3 of the 26 (11.5%) patients matched the physician on the first attempt. However, the feedback app enabled 21 of 26 (81%) volunteers to match the physician specified palpation to within 20% of the time and amplitude of the physician (Fig. 5b). These patients needed an average of 6 palpations to match the physician (Fig. 5c), with two patients requiring over 20 palpations. As each palpation takes approximately 8 seconds, testing was very quick and convenient. The patients that were unsuccessful in matching the physician had either highly unorthodox palpation shapes or consistently low palpation amplitudes that the supervising physician would observe.

With a sensitivity and specificity of 95%, the app provides excellent and accurate feedback to the patient. In the current state, the patient attempts to match the physician by performing nine palpations. Should no match to the physician palpation occur, the patient may attempt the set of nine palpations three more times, for a total of 36 palpations. Here, the sensitivity and specificity were calculated by using the outcome from each set of nine palpations (n = 57), either matching or not matching (Fig. 5d). Furthermore, both false positive and negative outcomes were often identified quickly as they were typified by a highly unorthodox shape. This enables the supervising physician to focus on the patient’s overall health and response to each palpation, rather than carefully scrutinizing large amounts of data.

## Discussion

Taken together, our data shows that smartphones may be used measure, compare, and match palpation motions from different, geographically separated, individuals. Both medically and non-medically trained individuals successfully palpated with a smartphone. This implementation was purposefully reductionist in nature and determined that a single accelerometer sufficiently measures palpations. However, using the integrated multi-axis accelerometers, gyroscopes, and more sophisticated matching algorithms will further improve the accuracy of the system and reduce error. The high sensitivity and specificity (95%) of the feedback application minimizes both patient and physician effort. For patients, the feedback enables the vast majority of individuals (81%) to match the physician specified palpation in just six attempts, which equates to just a minute or two of time. Similarly, physicians may focus on gauging the patient’s response to each palpation and provide guidance on proper palpation technique, instead of intensively scrutinizing data. In consideration of how long an average trip to the clinic takes, this approach could represent a remarkable convenience and time-savings for a near zero cost. In light of these initial results, large studies are being planned to assess the utility of this approach in the clinical setting. While this study explored the use of a 20% acceptance window in the feedback application, this number will likely change during the course of the clinical study, which will seek to define what acceptance window is sufficient for screening of abdominal pain.

The physician will always need to review the quantitative data on a patient’s motion to ensure that it meets his or her criteria for a useful palpation. Since this technology is designed to assist with triage of acute abdominal pain, the physician will be seeking evidence that the patient is not in imminent danger. While the app enables a patient to recapitulate a physician’s motion, the system may still have a 5% error due to small rotations of the smartphone during a palpation. To simplify the use of the app for the patient, the acceptance criteria only includes the peak time and magnitude, not the width of the peak. Hence, the physician will need to incorporate the quantitative information from the palpation into other clinically relevant information gathered from the history and visual observation of the patient to judge the patient’ overall condition (Fig. 1). A physician may always ask a patient to repeat palpations should he or she feel that additional data and evidence would be useful in evaluating the patient. If the physician is unable to provide clear and convincing evidence that the patient is not in a critical condition, he or she can refer them to seek urgent/emergency care immediately. However, if we assume that physicians are able to successful identify the vast majority of non-emergency cases, we can provide an estimate for the total number of saved emergency department visits. Noting that 9% of all emergency room visits are for non-emergency abdominal pain, and that 81% of patients were able to palpate remotely, we estimate that this technology could redirect up to 9.1 million emergency department visits in the United States alone^21^. However, a full clinical assessment study is needed to validate this approach.

Enabling remote triage will help to better allocate limited resources as well as improve the access and convenience of care for patients. Since one only needs a smartphone, this tangible benefit comes at almost no cost and represents an exceptional value to the healthcare community. This technique may also leverage the extensive cellular networks and availability of smartphones in developing countries to provide cost-effective and accessible healthcare. Remote screening and assessment in developing countries would help better distribute the limited resources in a timely manner. Our technology is a first step towards touch capable telemedicine and represents a new paradigm in a mHealth technologies, which are radically changing the practice of medicine. We envision that our work towards enabling a remote is a part of a future where the vast majority of medical care begins at home with a virtual visit to a physician who is able to remotely perform all routine examinations.

Our survey data indicates that physicians seek to use consistent palpation styles, but due to a lack of technology, they receive no feedback on their actual motion. For example, although our data shows that while a physician may perform consistent palpations (Supplemental Fig. 3), due to an absence of feedback, the physician examined in this study switched between two different palpation styles. This system may help physicians to be more consistent by now providing feedback on each palpation. By helping them to associate tactile feeling and sensory input with quantitative numbers, the physicians would be able to adjust subsequent palpations and “self-calibrate” to achieve a desired motion. Such a system could also be very useful in clinician education and training, where students and trainees learn palpation technique through an apprentice-based approach involving observation and extensive practice with an experienced physician. This interaction could be enhanced by enabling instructors to quantitatively measure how a trainee’s palpation compares to their own. In addition, enabling the measurement of each physician’s palpation enables comparison between palpations and new testing to determine whether an optimal palpation style exists, as defined by better diagnosis.

## Methods

### Phone and Accelerometer Data Collection

All testing was performed with a Samsung Galaxy S5 running the Android 4.4.2 operating system. The Android operating system was chosen since implementation of new applications is more expedient, but similar results would be expected for Apple’s iOS and other operating systems. As the goal of this study was to test and validate the use of a smartphone to measure and recapitulate palpations, all volunteers used the same phone for testing. Future studies will assess if and whether phone form factor introduces errors.

One important consideration for Android systems is that acceleration data is only collected when the current acceleration differs from the previous measurement by a certain threshold. As such it was unclear as to whether acceleration would be continuously or intermittently measured, and possibly affect the measurement of palpation time. Preliminary experiments showed that acceleration was continuously recorded due to a combination of: a low set threshold, a high accelerometer sensitivity, and the inability of an individual to hold their non-resting hand at zero acceleration. Even when an individual attempted to hold a phone “still”, the small natural movements that accompany appendage stabilization were measured and above the threshold for data collection. Hence, a person holding a phone has a natural acceleration “noise”, effectively ensuring that data is continually collected, minimizing time errors.

### Consistency Testing with Abdominal Model

For initial phone characterization, testing, and algorithm development, and abdominal analog was constructed. The goal of this testing apparatus was to create a system for the initial characterization of the smartphone and to demonstrate proof of principle viability of this approach. There were two primary reasons that a testing apparatus was chosen over testing in human subjects. First, there will always be some degree of variability in the palpation curves of the users, both patients and to a lesser extent, physicians, as demonstrated by our data. Second, there is variability in the mechanical properties of the abdomen itself (intra- and inter-subject variability) as the abdomen is not an isotropic material and palpation itself may shift and displace abdominal contents. Instead, a system was needed in which the user motion and the “abdomen” were constant. Here, upholstery foam was used to qualitatively simulate the physiological stiffness of an abdomen on a flat expansive surface conducive to testing. When combined with a servo to provide repeatable motions, which qualitatively match physician palpations, this system facilitated the characterization of the smartphone motion sensing as well as algorithm development.

An SPG5685A-CM-360 channel mount Servo Power Gearbox with a 7:1 gear ratio (RobotZone LLC, Winfield KS) was used to move the phone in a repeatable manner. The linkage used to hold the phone was constructed from 6–32 hexagon aluminum standoffs, 2 inches (50.8 mm) in length, and a 1/16″ thick (1.6 mm) aluminum bar designed to keep the standoffs 6.5 cm apart. Servo speeds and depths of compressions were informed by observations of several physicians palpating. To quantify the extent of the range of the technique, speeds and depths of compressions were visually estimated to be higher and lower than that of a physician. However, later experimental data demonstrated that the servo speed and compression depth did not accurately recapitulate the magnitude of the peak acceleration used by a physician, but instead was smaller than anticipated. In this case the data remains valid, as it demonstrates a high repeatability and sensitivity to a smaller magnitude of acceleration than what is typically used by individuals. Hence, the larger acceleration values used by individuals were of sufficient magnitude to be easily measured in a repeatable fashion.

In our system, palpation motions are typically described in terms of position and velocity, whereas the data presented is in terms of acceleration. Since the phone-abdomen system may be modeled as a mass-spring system to which a force is applied, the velocity and acceleration are first and second order derivatives of the position, respectively. Hence, the position, velocity, and acceleration are inter-related and measuring any one of these quantities will be sufficient to define the motion of the object. Of these three related variables, microelectromechanical sensors may directly measure acceleration, so for ease, this variable was used for measurement and analysis. However, while measurements of acceleration are straightforward they are not necessarily intuitive for an individual performing an abdominal palpation, and hence motion and subsequent feedback are described in terms of depth (position) and speed (velocity).

### Algorithm and application development

Raw data was imported from the phone and imported into MATLAB (Mathworks, Inc). Refinement of data was performed in the following sequential order: remove offsets, remove zero values, moving average filter, and rotation correction (Supplemental Fig. 2). The moving average filter reduced the noise while retaining a sharp step response. Since each palpation is performed in a continuous fashion over a 1–2 s timeframe with a sampling interval of 0.0025 s, using the previous 30 time points for an averaging interval represented a good compromise between filtering noise and maintaining signal fidelity. The main source of error with this approach would be the loss of information when applying the filter to sudden stepwise changes in acceleration that occur over timeframes comparable to the sampling interval. In this experiment, such motions are deemed noise given the duration and continuous nature of a palpation.

The application was programmed in Java (Oracle Corp) for use on Samsung Galaxy S5 running the Android 4.4.2 operating system. The application on the smartphone provided auditory beeps to let the individual know when the measurement begins and ends. Acceleration curves were processed by MATLAB on an adjoining desktop PC, which was attached to the phone via mini-USB. In cases where feedback was used, simple instructions were displayed to the individual on the desktop PC after each palpation.

### Volunteer testing

Volunteers were consented and tested with a protocol approved by Georgia Tech’s Institutional Review Board and all experiments were performed in accordance with relevant guidelines. After obtaining informed consent for study participation, volunteers were asked to sit upright in a neutral position with feet flat on the floor. The individual proctoring the experiment demonstrated the proper anatomical location to hold the smartphone, and then handed the phone to the volunteer. Patients were also provided with verbal instructions on the proper use and palpation technique, but did not receive feedback on their individual palpation style. The volunteer launched the app on the phone, placed it on their abdomen and listened for an audible beep, indicating that they should begin the compression. A second beep indicated that data collection was completed. In cases where feedback testing was performed, feedback was provided to the volunteer on a computer screen indicating how well the compression matched the target compression. All acceleration data was recorded.

### Data Availability

The datasets generated during and/or analysed during the current study are available from the corresponding author on reasonable request.

## Electronic supplementary material

Supplementary Information
