# Supplementary material for: Towards remote assessment and screening of acute abdominal pain using only a smartphone with native accelerometers
Source: Sci Rep. 2017 Oct 6;7:12750. doi: 10.1038/s41598-017-13076-x (PMC5630621; doi:10.1038/s41598-017-13076-x)
Supplement: Supplementary file 1 — Supplementary Information [file 41598_2017_13076_MOESM1_ESM.pdf]

Towards remote assessment and screening of acute abdominal pain using only a smartphone with native accelerometers

David R. Myers, PhD[1,2,3,4,5+], Alexander Weiss[1+], Margo Rollins, MD [1,2,3,4,5],
Wilbur A. Lam, MD, PhD[*,1,2,3,4,5]

[1] The Wallace H. Coulter Department of Biomedical Engineering, Georgia Institute of Technology & Emory University, Atlanta, GA, 30332
[2] Department of Pediatrics, Division of Pediatric Hematology/Oncology, Aflac Cancer Center and Blood Disorders Service of Children's Healthcare of Atlanta, Emory University School of Medicine, Atlanta, GA 30322
[3] Winship Cancer Institute of Emory University, Atlanta, GA, 30322
[4] Parker H. Petit Institute of Bioengineering and Bioscience, Georgia Institute of Technology, Atlanta, GA 30332
[5] Institute for Electronics and Nanotechnology, Georgia Institute of Technology, Atlanta, GA 30332
+ These authors contributed equally to this work.
* Corresponding author: wilbur.lam@emory.edu

Supplementary Figures

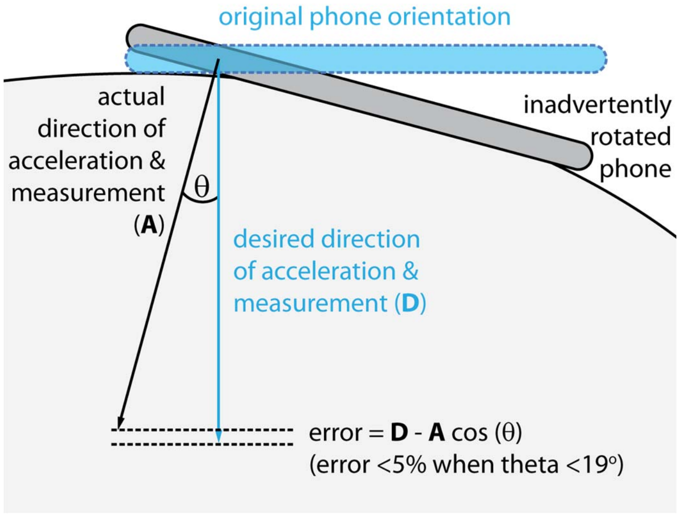

Supplemental Figure 1: An analytical model shows that significant tilts are associated with low errors in measuring the acceleration. By placing the phone on the abdomen before beginning the measurement, the orientation is set and normalized by anatomical features. Should the user rotate the phone during the palpation, this model estimates that there will be a 5% error in the desired direction of acceleration (D) when the phone is rotated 19 degrees. Hence, for this particular application, small rotations (<19 degrees) will introduce minimal error, whereas larger rotations will be visible to the supervising physician who can ask the patient to repeat the test.

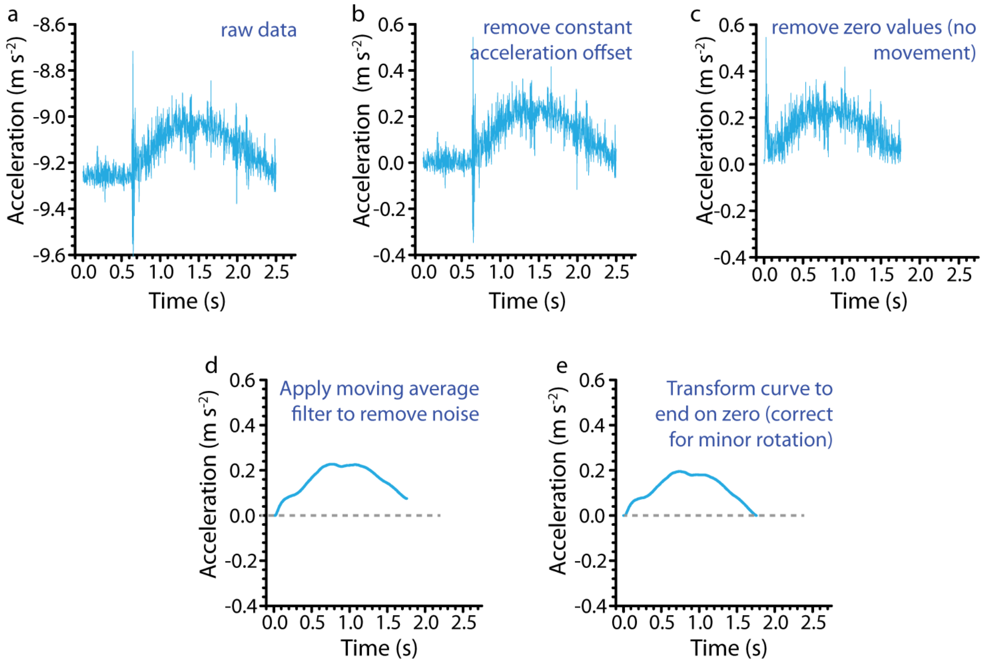

Supplemental Figure 2: Raw smartphone acceleration is filtered to enable simple comparisons of different palpations. Here, an example curve taken from the testing apparatus is shown to illustrate the filtering operations. a, The raw acceleration data is noisy and offset. This complicates the comparison of the duration and magnitude of different palpations. Here, pre-filtering of the signal is used to simplify comparisons. b, In the testing apparatus, the measurement axis and is aligned with gravity, creating a large offset which is removed. c, The data may also include measurements of no movement. Since it is important to measure the duration of the palpation, these zero values are also removed, and the graph is shifted such that the estimated beginning of a palpation occurs at time zero. e, A moving average filter is next used to remove the noise. f, Each signal should ideally start and stop at zero acceleration, but since a single axis is used for measurements, minor changes to the rotation of the phone will introduce offsets between the start and stop acceleration. In this case, the measurement is assumed to be a superposition of the small acceleration induced by rotating the phone and the acceleration induced by the compression. Here, the data is transformed and this offset from rotation is removed to enable more accurate comparisons.

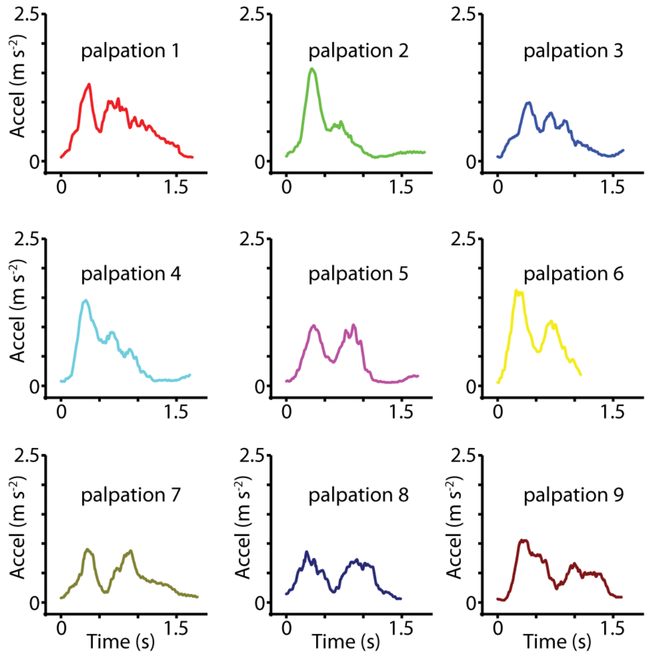

Supplemental Figure 3: A single physician repeating palpations may use varied compression styles but with broadly similar values unique to that person. Here, a physician repeatedly self-palpated using the smartphone. The curves indicate that the physician switched between two different palpation styles, but retained a measure of consistency as measured by the peak magnitude and standard deviation in either case. Palpations 2,4, and 6 have a distinct increase in acceleration to a maximum value (average 1.55 m/s^2, standard deviation 0.071 m/s^2) with a subsequent decrease to rest. Alternatively, palpations 3,5,7,8, and 9 have multiple defined peaks during the same time frame, with a similar maximum peak magnitude (average 0.989 m/s^2, standard deviation 0.079 m/s^2).

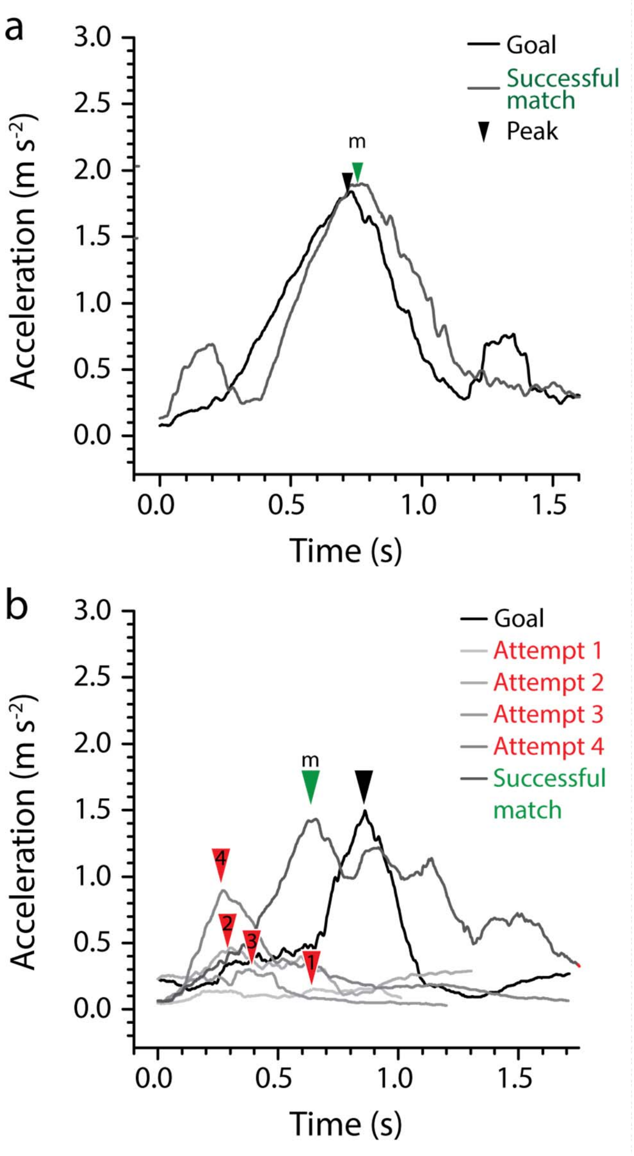

Supplemental Figure 4: Feedback enables patients to match themselves. The feedback algorithm compares the time from rest to peak acceleration as well as the magnitude of the peak acceleration. **a**, Some patients exhibit highly repeatable palpations, such as the one shown here who matched their own palpation on the first try. **b**, Other patients exhibit difficulty matching their own palpation, shown here, but were able to successfully match using the feedback algorithm. However, while the peaks are within 20% of the magnitude or time, the match has a less pronounced release than the original target, highlighting the need for a physician interpretation of the data.
